# Reperfusion by endovascular thrombectomy and early cerebral edema in anterior circulation stroke: Results from the SITS-International Stroke Thrombectomy Registry

**DOI:** 10.1177/17474930231180451

**Published:** 2023-06-17

**Authors:** Magnus Thorén, Irene Escudero-Martínez, Tomas Andersson, Shih-Yin Chen, Nicole Tsao, Dheeraj Khurana, Simone Beretta, Andre Peeters, Georgios Tsivgoulis, Christine Roffe, Niaz Ahmed

**Affiliations:** 1 Stroke Research Unit, Department of Clinical Neuroscience, Karolinska Institutet, Stockholm, Sweden; 2 Department of Neurology, Danderyd Hospital, Stockholm, Sweden; 3 Department of Neurology, Hospital Universitari i Politécnic La Fe, Valencia, Spain; 4 Neurovascular Research Laboratory, Instituto de Biomedicina de Sevilla (IBiS), Sevilla, Spain; 5 Institute of Environmental Medicine, Karolinska Institutet, Stockholm, Sweden; 6 Value & Access, Biogen, Cambridge, MA, USA; 7 Global Medical Affairs, Biogen, Cambridge, MA, USA; 8 Department of Neurology, Postgraduate Institute of Medical Education and Research, Chandigarh, India; 9 Department of Neurology and Stroke Unit, San Gerardo Hospital, Monza, Italy; 10 Department of Neurology and Stroke Unit, Cliniques Universitaires Saint-Luc, Brussels, Belgium; 11 Second Department of Neurology, National & Kapodistrian University of Athens, Athens, Greece; 12 Stroke Research, Keele University, Stoke-on-Trent, UK; 13 Department of Neurology, Karolinska University Hospital, Stockholm, Sweden

**Keywords:** Ischaemic stroke, cerebral edema, reperfusion, thrombolysis, outcome, intracerebral hemorrhage, cerebral infarction

## Abstract

**Background:**

A large infarct and expanding cerebral edema (CED) due to a middle cerebral artery occlusion confers a 70% mortality unless treated surgically. There is still conflicting evidence whether reperfusion is associated with a lower risk for CED in acute ischemic stroke.

**Aim:**

To investigate the association of reperfusion with development of early CED after stroke thrombectomy.

**Methods:**

From the SITS-International Stroke Thrombectomy Registry, we selected patients with occlusion of the intracranial internal carotid or middle cerebral artery (M1 or M2). Successful reperfusion was defined as mTICI ⩾ 2b. Primary outcome was moderate or severe CED, defined as focal brain swelling ⩾1/3 of the hemisphere on imaging scans at 24 h. We used regression methods while adjusting for baseline variables. Effect modification by severe early neurological deficits, as indicators of large infarct at baseline and at 24 h, were explored.

**Results:**

In total, 4640 patients, median age 70 years and median National Institutes of Health Stroke Score (NIHSS) 16, were included. Of these, 86% had successful reperfusion. Moderate or severe CED was less frequent among patients who had reperfusion compared to patients without reperfusion: 12.5% versus 29.6%, p < 0.05, crude risk ratio (RR) 0.42 (95% confidence interval (CI): 0.37–0.49), and adjusted RR 0.50 (95% CI: 0.44-0.57). Analysis of effect modification indicated that severe neurological deficits weakened the association between reperfusion and lower risk of CED. The RR reduction was less favorable in patients with severe neurological deficits, defined as NIHSS score 15 or more at baseline and at 24 h, used as an indicator for larger infarction.

**Conclusion:**

In patients with large artery anterior circulation occlusion stroke who underwent thrombectomy, successful reperfusion was associated with approximately 50% lower risk for early CED. Severe neurological deficit at baseline seems to be a predictor for moderate or severe CED also in patients with successful reperfusion by thrombectomy.

## Introduction

Cerebral edema (CED) in acute ischemic stroke, caused by transvascular flow of plasma over damaged blood–brain barrier, worsens the prognosis and may cause life-threatening intracranial tissue shifts.^1,2^ A major predictor for the extent and radiological severity of the edema is infarct size. Other risk factors, some of which may be related to infarct size, for CED in patients with extensive ischemia include high blood glucose, signs of acute infarct, hyperdense artery on baseline scans, and decreased level of consciousness.^3,4^ In anterior circulation large artery occlusion, the risk of life-threatening CED is highest in subtotal or complete middle cerebral artery (MCA) infarction.^5^ More than one-third of patients with large MCA infarctions clinically deteriorate within 24 h and two-thirds deteriorate within 48 h of stroke onset.^6,7^ The mortality is 70% within a few days, unless treated with early decompressive surgery.^8^

There is conflicting evidence regarding the effect of reperfusion on edema. Some data indicated an increased risk of severe edema in reperfused brain tissues.^9,10^ Recent studies, however, indicate that reperfusion decreases the risk of edema.^11–13^ In a meta-analysis of thrombectomy data, there was no association between reperfusion and imaging signs of edema, except in patients with large ischemic core volume where increased midline shift was detected.^14^ Using data from the SITS-International Stroke Thrombolysis and/or Thrombectomy Registry, we found that signs of recanalization, detected mostly by non-contrast imaging, was associated with a lower risk for early edema, and a higher adjusted risk for parenchymal hematoma (PH), in a cohort of ischemic stroke patients where more than 80% had received only intravenous thrombolysis (IVT) treatment.^15^ Since then, the SITS Registry has included more data from patients undergoing thrombectomy. We hypothesized that, using these detailed angiographic data from patients undergoing thrombectomy, reperfusion would decrease the risk for CED in patients with large artery occlusion stroke. Moreover, we aimed to consider the potentially mediating effect of PH since reperfusion seemed to be positively associated with PH.^15^ In addition, PH alone may provoke brain swelling.

## Aims

The primary aim was to investigate the effect of reperfusion on the risk for moderate or severe CED early (at 24 h) after thrombectomy in anterior circulation large artery occlusion stroke including adjustment for PH. A secondary aim was to investigate the modifying effect of indicators of infarct size at baseline.

## Methods

The SITS-International Stroke Thrombectomy Registry (ISTR) is an Internet-based academic interactive, prospective register for the monitoring of treatment in acute ischemic stroke. All data were collected by local investigators. Methods of data collection in SITS-ISTR have been described in detail elsewhere.^16–18^ From SITS-ISTR, we included patients with data entered according to the Standard Thrombectomy data entry protocol 2014–2019 that had angiographically verified occlusion of the intracranial internal carotid artery or MCA (M1 or M2 segments) and had undergone thrombectomy. We included centers with at least 10 patients recruited and at least a 70% follow-up rate at 3 months. Patients were excluded if the data were incomplete for age, reperfusion status, or results from brain imaging at 24 h.

Reperfusion status was rated at the end of the thrombectomy procedure using the modified Treatment in Cerebral Ischemia (mTICI) score.^19^ In the primary analysis, reperfusion status dichotomized into successful reperfusion (mTICI 2b or 3) versus non-reperfusion (mTICI ⩽ 2a). In a secondary analysis, reperfusion status was treated as an ordinal variable with four levels (mTICI 0 or 1, 2a, 2b, 3). Post-thrombectomy CED was rated at 24 (allowed interval 22–36) h using the SITS-MOST edema scale where mild CED is defined as focal brain swelling up to one-third of the hemisphere, moderate CED as focal brain swelling greater than one-third of the hemisphere, and severe CED as focal brain swelling with midline shift.^4,15,20,21^ During analysis, the CED variable was either dichotomized into moderate or severe CED versus no or mild, or used as variable with four levels. PH at 24 h was defined as any hemorrhage of the infarct area with mild or substantial space-occupying effect, that is, PH of either type 1 or type 2, respectively.^22^

Other variables extracted for analysis included baseline characteristics, neurological severity as measured by National Institutes of Health Stroke Scale (NIHSS) score at baseline and at 24 h, computed tomography (CT) or magnetic resonance imaging (MRI) infarct signs on first examination, pre-morbid functional status as measured by modified Rankin Scale (mRS), pre-existing conditions, medication history, IVT, time from stroke onset to the end of the thrombectomy procedure and whether the patient was treated in a stroke unit, right- or left-sided vascular involvement, and functional outcome as measured by mRS score and death at 3-month follow-up. When reporting this study, we adhered to the STROBE observational cohort guideline.^23^

### Statistical methods

In order to estimate risk ratios (RR) with 95% confidence intervals of moderate or severe CED in relation to explanatory variables and for purposes of statistical modeling, we used generalized linear regression with log link. Continuous variables were categorized into quartiles as needed. Missing values in categorical covariates were included as a separate level in that variable, missing values in continuous covariates were set to 0 and the variable was extended with a binary indicator for missing value. Likelihood ratio tests were used in model building and variable selection. Selection of adjustment variables was done with the model for successful reperfusion using dichotomized CED and a procedure of backward stepwise elimination (variables with p < 0.05 were retained in the final model). The starting model for this procedure contained all variables independently associated with CED in univariable analyses, excluding heparin treatment and decompressive hemicraniectomy due to extremely low number of events. In addition, reperfusion status, age, sex, baseline NIHSS score, and baseline glucose were introduced and kept in all models, regardless of results from analyses. To facilitate analysis, pre-morbid mRS was dichotomized into 0–1 versus 3–5 and time to end of thrombectomy was categorized into quartiles. The final model was then used for exploratory analyses. Any indirect (mediating) effect of intracerebral hemorrhage on edema was explored by adding PH. We also explored the effect modification of the RR between reperfusion and CED by factors potentially related to infarct size: severe hemispheric syndrome (SHS), defined as NIH Stroke Scale score 15 or more at baseline, and persistent severe hemispheric syndrome (P-SHS), defined as NIH Stroke Scale score 15 or more at 24 h. Previously selected adjustment variables were also used in models for the effect of each specific CED level versus “no CED.” Functional outcome using mRS and mortality were calculated based on reperfusion status. Two-tailed p values less than 0.05 were regarded as statistically significant.

### Ethics

Ethics approval was obtained from the Stockholm Regional Ethics Committee for this project as part of the SITS-MOST II study framework (Dnr 2022-01157-02). Ethics approval and patient consent for participation in the SITS-ISTR were obtained in countries where required; remaining countries approved the register for anonymized audit.

## Results

**Figure 1.**
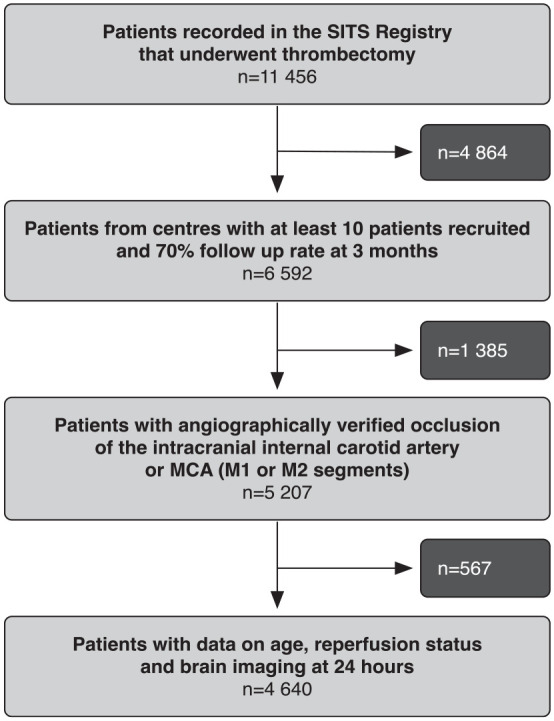
Flowchart of the study.

In total, 4640 patients, were included into the study cohort. Figure 1 depicts the selection procedure. Median age of the study cohort was 70 years with a median NIHSS score of 16. As seen in Table 1, successful reperfusion was achieved in 86% of patients. Moderate or severe CED at 24 h was detected in 693 (18%) of patients. Compared to patients with no or mild CED, patients with moderate or severe CED had higher baseline NIHSS (mean 18.4 vs 15.4), more frequent early CT or MRI signs of infarction (49% vs 29%), slightly higher plasma glucose, and a higher prevalence of diabetes (24% vs 20%). Moderate or severe CED was less frequent among patients that had successful reperfusion compared to patients without reperfusion: 12.5% versus 29.6%, p < 0.05, crude RR (cRR) 0.42 (95% CI: 0.37–0.49). Univariable risks for moderate or severe CED, by individual characteristics, are shown in the online supplemental material.

**Table 1.** Descriptive statistics of the cohort.

Characteristic	All		No or mild CED at 24 h		Moderate or severe CED at 24 h		p
	n	Mean (SD) or %	n	Mean (SD) or %	n	Mean (SD) or %	
Successful reperfusion (%)	4640	85.6	3476	88.1	495	71.4	<0.05
mTICI (%)							<0.05
0–1	336	7.2	240	6.1	96	13.9	
2a	333	7.2	231	5.9	192	14.7	
2b	1245	26.8	1039	26.3	206	29.7	
3	2726	58.8	2437	61.7	289	41.7	
Age, years (mean)	4640	70.2 (13.7)	3947	70.4 (13.7)	693	69.1 (13.8)	<0.05
Male sex (%)	4640	49.4	3947	49.3	693	50.1	0.70
NIHSS baseline (mean)	4541	15.8 (6.0)	3868	15.4 (6.0)	673	18.4 (5.2)	<0.05
NIHSS score ⩾15 at baseline (SHS) (%)	4541	55.8	3868	52.4	673	74.9	<0.05
NIHSS score ⩾15 at 24 h (P-SHS) (%)	4168	28.7	3619	22.2	549	71.2	<0.05
Early signs of infarction (%)	4612	32.3	3927	29.3	685	49.3	<0.05
Mean arterial pressure at baseline, mmHg (mean)	4214	103 (16)	3588	103 (16)	626	105 (18)	<0.05
Plasma glucose at baseline, mmol/L (mean)	3991	7.4 (4.5)	3384	7.3 (4.7)	607	8.0 (3.0)	<0.05
Plasma cholesterol at baseline, mmol/L (mean)	2665	4.7 (6.1)	2347	4.7 (6.4)	318	4.5 (1.2)	0.48
Pre-morbid mRS (%)							<0.05
0	3591	77.9	3094	78.8	497	72.4	
1	430	9.3	339	8.6	91	13.3	
2	296	6.4	243	6.2	53	7.7	
3	199	4.3	164	4.2	35	5.1	
4	86	1.9	77	2.0	9	1.3	
5	8	0.2	7	0.2	1	0.1	
Previous stroke ⩾3 months earlier (%)	4616	9.7	3928	9.9	688	8.9	0.40
Previous stroke <3 months earlier (%)	4620	2.0	3930	2.0	690	2.2	0.71
Previous TIA (%)	4615	3.3	3926	3.3	689	2.9	0.57
Atrial fibrillation (%)	4611	31.2	3924	31.3	687	30.6	0.68
Diabetes mellitus (%)	4621	20.2	3931	19.5	690	24.3	<0.05
Congestive heart failure (%)	4610	10.4	3922	10.7	688	8.9	0.14
Hypertension (%)	4623	68.5	3932	68.6	691	67.9	0.69
Hyperlipidemia (%)	4603	36.2	3916	35.7	687	39.2	0.08
Smoker (%)							0.78
Never	3403	77.1	2903	77.2	500	76.6	
Previous	372	8.4	319	8.5	53	8.1	
Current	639	14.5	539	14.3	100	15.3	
Aspirin treatment (%)	4589	25.5	3909	25.5	680	25.4	0.99
Clopidogrel treatment (%)	4594	5.7	3913	5.6	681	6.2	0.53
Other antiplatelet treatment (%)	4595	1.3	3913	1.4	682	0.9	0.31
Statin treatment (%)	4577	31.3	3899	30.8	678	34.4	0.06
Oral hypertensive medication (%)	4585	66.4	3906	67.0	679	63.2	0.05
Oral antidiabetic medication (%)	4596	13.5	3915	12.7	681	17.6	<0.05
Insulin treatment (%)	4597	4.1	3915	4.0	682	4.5	0.54
IVT (%)	4280	61.3	3947	62.2	693	56.1	<0.05
Stroke onset to end of thrombectomy, min (mean)	4018	314 (165)	3434	310 (166)	584	336 (157)	<0.05
Stroke unit care (%)	4640	83.9	3947	84.4	693	81.5	0.06
Vascular territory (%)							<0.05
Left	2245	52.2	1921	52.8	324	48.8	
Right	2019	46.9	1691	46.5	328	49.4	
Bilateral	37	0.9	25	0.7	12	1.8	

Chi-square test or t test was used as appropriate.

Table 2 shows risks for moderate or severe CED resulting from the adjusted models. In patients with successful reperfusion, adjusted RR (aRR) for moderate or severe CED was 0.50 (0.44–0.57) with an area under the curve (AUC) of 0.75. Compared to patients with mTICI 0 or 1, aRR for moderate or severe CED in patients with progressively higher mTICI grades were: for mTICI 2a, aRR 1.24 (1.01–1.54); for mTICI 2b, aRR 0.67 (0.55–0.82); and for mTICI 3, aRR 0.49 (0.41–0.60) with AUC 0.75. In successfully reperfused patients, adding PH to the model increased aRR from 0.50 to 0.63 (0.55–0.72), indicating that about one-fourth of the protective effect of successful reperfusion was lost. Meanwhile, successfully reperfused patients had less parenchymal hemorrhage compared to non-reperfused (8.6% vs 12.7%, p < 0.05). To demonstrate the extent of effect modification, separate models were run stratifying by SHS and P-SHS. As seen in Table 3, and presented in detail in the online supplemental material, the aRR for CED with reperfusion was 0.32 (0.24–0.42) among patients without SHS and 0.56 (0.48–0.66) among patients with SHS with corresponding aRR for P-SHS 0.36 (0.25–0.53) and 0.81 (0.69–0.95). This indicated that the association between reperfusion and lower risk of CED, interpreted as the protective effect of successful reperfusion, was weakened by the presence of SHS or P-SHS. A model for CED with four levels is shown in the online supplemental material.

**Table 2.** Final adjusted models for moderate or severe CED.

Parameters remaining after elimination	Moderate or severe CED			
	Reperfusion classified into successful versus unsuccessful^a^		Reperfusion classified into ordinal mTICI categories^b^	
	RR (95 % CI)^c^	p^d^	RR (95 % CI)^c^	p^d^
Succesful reperfusion	0.50 (0.44–0.57)	<0.05		
mTICI				
0–1			Reference	<0.05
2a			1.24 (1.01–1.54)	
2b			0.67 (0.55–0.82)	
3			0.49 (0.41–0.60)	
Age, years	0.99 (0.98–0.99)	<0.05	0.99 (0.99–0.99)	<0.05
Male sex	0.97 (0.86–1.11)	0.74	1.00 (0.88–1.14)	0.99
Oral antidiabetic medication	1.33 (1.13–1.59)	<0.05	1.29 (1.10–1.52)	<0.05
NIHSS baseline	1.07 (1.06–1.09)	<0.05	1.08 (1.07–1.09)	<0.05
Early signs of infarction	1.70 (1.49–1.93)	<0.05	1.65 (1.45–1.87)	<0.05
Mean arterial pressure at baseline, mm Hg	1.00 (1.00–1.01)	<0.05	1.01 (1.00–1.01)	<0.05
Plasma glucose at baseline, mmol/L	1.04 (1.02–1.05)	<0.05	1.04 (1.02–1.06)	<0.05
Stroke onset to end of thrombectomy, min				
⩽212	Reference	<0.05	Reference	<0.05
213–280	1.35 (1.07–1.71)		1.37 (1.08–1.73)	
281–370	1.32 (1.05–1.65)		1.32 (1.06–1.66)	
⩾370	1.54 (1.23–1.92)		1.55 (1.24–1.94)	
Vascular territory				
Left	Reference	<0.05	Reference	<0.05
Right	1.36 (1.19–1.55)		1.41 (1.23–1.61)	
Bilateral	2.15 (1.40–3.30)		2.13 (1.38–3.30)	

a Model AUC 0.75.

b Model AUC 0.75.

c Wald’s method.

d LR test.

**Table 3.** Demonstration of effect modification by potential indicators of large infarct, SHS (severe hemispheric syndrome) at baseline, and P-SHS (persistent severe hemispheric syndrome) at 24 h using the final adjusted model.

Time of examination of NIHSS score	Sample included in model, n	RR (95 % CI) for moderate or severe CED in patients with succesful reperfusion versus patients without succesful reperfusion	
		NIHSS under 15	NIHSS 15 or more
Baseline (SHS)	4541	0.32 (0.24–0.42)	0.56 (0.48–0.66)
24 h (P-SHS)	4168	0.36 (0.25–0.53)	0.81 (0.69–0.95)

Functional outcome, as assessed by mRS score, and mortality at 3 months were better among reperfused versus non-reperfused patients. Among reperfused patients, 65.1% achieved mRS 3 or better, compared to 33.4% of non-reperfused patients. In addition, mortality was lower in reperfused patients compared to non-reperfused patients (16.2% vs 36.5%).

## Discussion

In this multinational study of patients with anterior circulation large artery occlusion stroke who were treated with thrombectomy and received subsequent radiological evaluation for early CED at 24 h, increasing degree of reperfusion was associated with lower rates of early CED. In particular, successful reperfusion (mTICI 2b or better) was associated with approximately 50% lower risk for early moderate or severe CED (aRR: 0.50). The results were consistent using different statistical approaches. The risk reduction was less favorable in patients with severe neurological deficits which may indicate that large early infarcts confer a higher risk for early CED despite successful reperfusion. Reperfusion resulted in a better functional outcome and lower mortality within the first 3 months.

This study strengthens the evidence that reperfusion decreases the risk for early CED in large artery occlusion stroke. This is consistent with recent clinical studies that used different methods to detect CED.^11–15^ Net water uptake quantified using CT scan, which despite a relative lack of validation has been used as a biomarker of CED, was significantly reduced in patients with vessel recanalization.^24,25^ In contrast to our previous study,^15^ successfully reperfused patients in this study had a lower incidence of PH compared to non-reperfused patients. Adjustment for PH increased the aRR for moderate or severe CED in reperfused versus non-reperfused patients from 0.50 to 0.63. This is consistent with about one-fourth of the protective effect of successful reperfusion being mediated by PH. Furthermore, and taking into consideration the benefit of thrombectomy over medical management shown in recent trials in patients with large ischemic core,^26–29^ our study adds evidence that the size of infarction is a determinant of risk for early CED even in patients with successful reperfusion. In statistical testing of effect modification of the association between successful reperfusion and lower risk of moderate or severe CED, SHS at baseline and P-SHS at 24 h were significant. These variables selected patients with large early clinical deficits, indicative of larger early ischemic core. The protective effect of reperfusion was attenuated more in patients with clinical signs of large early infarction at 24 h versus baseline. This result is consistent with a recent meta-analysis that investigated the effects of thrombectomy and reperfusion on CED and the impact of CED on functional outcome in patients presenting with P-SHS, defined by radiological signs of large infarction on pretreatment imaging as having an ischemic core volume of 80–300 mL. It was found that reperfusion was not associated with midline shift except in the subgroup with very large core volume (>130 mL).^14^

This study has some limitations, in addition to the inherent limitations due to the observational study design. First, as there was no central reading of imaging scans, the interpretation of the SITS edema scale may vary between investigators.^4,15,20,21^ In favor of the reliability of the SITS edema scale, however, several researchers have used similar imaging findings to classify swelling in cerebral infarcts.^15^ Regarding PH, there is potential for both underreporting and overreporting, the latter because contrast stains can be interpreted as small hemorrhages. However, the risk for overreporting is probably small because of the time interval to radiological examination which was performed approximately 24 h after stroke onset.^30^ Second, because of protocol, we were only able to detect early CED while later-developing CED would go undetected. Third, since there was no measurement of infarct volume, neurological severity was used as a proxy for infarct or core volume. Fourth, missing data was an issue; however, this was addressed as mentioned in section “Statistical methods.” The strengths of our study are the large sample size, the use of statistical methods that enabled us to estimate relative risks, the collection of data prospectively or at least in temporal proximity to actual events, the exactness of data on the site of arterial occlusion which enabled us to select patients with occlusion of the intracranial internal carotid artery or MCA, and the known time of reperfusion.

In conclusion, we observed that in patients with anterior circulation large artery occlusion stroke who were treated with thrombectomy, successful reperfusion was associated with a lower risk for radiologically detected CED at 24 h and this was consistent using different statistical approaches. Reperfusion conferred a decreased risk for PH. The relative risk reduction with reperfusion was less favorable in patients with severe hemispheric syndrome at baseline. Our results strengthen recent study results and should be considered in future trials for thrombectomy with signs of large ischemia.

## Supplemental Material

sj-docx-1-wso-10.1177_17474930231180451 – Supplemental material for Reperfusion by endovascular thrombectomy and early cerebral edema in anterior circulation stroke: Results from the SITS-International Stroke Thrombectomy Registry Click here for additional data file. Supplemental material, sj-docx-1-wso-10.1177_17474930231180451 for Reperfusion by endovascular thrombectomy and early cerebral edema in anterior circulation stroke: Results from the SITS-International Stroke Thrombectomy Registry by Magnus Thorén, Irene Escudero-Martínez, Tomas Andersson, Shih-Yin Chen, Nicole Tsao, Dheeraj Khurana, Simone Beretta, Andre Peeters, Georgios Tsivgoulis, Christine Roffe and Niaz Ahmed in International Journal of Stroke
