# Supplementary material for: Reperfusion by endovascular thrombectomy and early cerebral edema in anterior circulation stroke: Results from the SITS-International Stroke Thrombectomy Registry
Source: Int J Stroke. 2023 Jun 17;18(10):1193–201. doi: 10.1177/17474930231180451 (PMC10676032; doi:10.1177/17474930231180451)
Supplement: sj-docx-1-wso-10.1177_17474930231180451 – Supplemental material for Reperfusion by endovascular thrombectomy and early cerebral edema in anterior circulation stroke: Results from the SITS-International Stroke Thrombectomy Registry [file sj-docx-1-wso-10.1177_17474930231180451.docx]

**SUPPLEMENTAL MATERIAL**

**REPERFUSION BY ENDOVASCULAR THROMBECTOMY**

**AND EARLY CEREBRAL EDEMA IN ANTERIOR CIRCULATION STROKE:**

**RESULTS FROM SITS-ISTR**

Magnus Thorén, Irene Escudero-Martínez, Tomas Andersson, Shih-Yin Chen, Nicole Tsao, Dheeraj Khurana,  Simone Beretta, Andre Peeters, Georgios Tsivgoulis, Christine Roffe, Niaz Ahmed

List of supplemental tables

- Table I. Univariable risks for moderate or severe CED, by individual characteristics.
- Table II. Final models for CED levels (reference: no CED) with reperfusion classified into successful versus unsuccessful.
- Table III. Detailed data for the demonstration of effect modification by potential indicators of large infarct, using the final adjusted model.

**Table I**

Univariable risks for moderate or severe CED, by individual characteristics.

| Characteristic | Moderate or severe CED  versus no or mild CED | | |
| --- | --- | --- | --- |
|  | RR | 95% CI * | P † |
| Successful reperfusion | 0.42 | 0.37-0.49 | <.05 |
| mTICI |  |  | <.05 |
| 0-1 | Reference |  |  |
| 2a | 1.07 | 0.85-1.35 |  |
| 2b | 0.58 | 0.47-0.71 |  |
| 3 | 0.37 | 0.30-0.45 |  |
| Age, per year | 0.99 | 0.99-1.00 | <.05 |
| Male sex | 1.03 | 0.90-1.18 | .70 |
| NIHSS baseline, per point | 1.08 | 1.07-1.09 | <.05 |
| Early signs of infarction | 2.04 | 1.78-2.34 | <.05 |
| Mean arterial pressure at baseline, per 1 mmHg | 1.01 | 1.00-1.01 | <.05 |
| Plasma glucose at baseline, per 1 mmol | 1.06 | 1.05-1.08 | <.05 |
| Plasma cholesterol at baseline, per 1 mmol | 1.00 | 0.92-1.09 | <.05 |
| Pre-morbid mRS |  |  | <.05 |
| 0 | Reference |  |  |
| 1 | 1.53 | 1.25-1.87 |  |
| 2 | 1.29 | 1.00-1.67 |  |
| 3 | 1.27 | 0.93-1.74 |  |
| 4 | 0.76 | 0.41-1.41 |  |
| 5 | 0.90 | 0.14-5.66 |  |
| Previous stroke ≥ 3 months earlier | 0.9 | 0.71-1.16 | .53 |
| Previous stroke < 3 months earlier | 1.09 | 0.68-1.75 | .93 |
| Previous TIA | 0.89 | 0.59-1.35 | .84 |
| Atrial fibrillation | 0.97 | 0.83-1.13 | .65 |
| Diabetes mellitus | 1.27 | 1.08-1.49 | <.05 |
| Congestive heart failure | 0.84 | 0.65-1.07 | .32 |
| Hypertension | 0.97 | 0.84-1.12 | .86 |
| Hyperlipidemia | 1.13 | 0.98-1.30 | .22 |
| Smoker |  |  | .60 |
| Never | Reference |  |  |
| Previous | 0.97 | 0.75-1.26 |  |
| Current | 1.07 | 0.87-1.30 |  |
| Aspirin treatment | 1.00 | 0.85-1.17 | .14 |
| Clopidogrel treatment | 1.10 | 0.82-1.46 | .12 |
| Other antiplatelet treatment | 0.68 | 0.32-1.46 | .14 |
| Statin treatment | 1.15 | 0.99-1.33 | <.05 |
| Oral hypertensive medication | 0.87 | 0.75-1.00 | <.05 |
| Oral antidiabetic medication | 1.37 | 1.15-1.64 | <.05 |
| Insulin treatment | 1.11 | 0.80-1.54 | .16 |
| IVT | 0.81 | 0.70-0.93 | <.05 |
| Stroke onset to end of thrombectomy, minutes |  |  | <.05 |
| ≤212 | Reference |  |  |
| 213-280 | 1.43 | 1.12-1.82 |  |
| 281-370 | 1.62 | 1.28-2.05 |  |
| ≥370 | 2.00 | 1.59-2.51 |  |
| Stroke unit care | 0.84 | 0.71-1.01 | .06 |
| Vascular territory (%) |  |  | <.05 |
| Left | Reference |  |  |
| Right | 1.13 | 0.98-1.30 |  |
| Bilateral | 2.25 | 1.40-3.62 |  |

* Wald´s method

† LR test

**Table II**

Final models for CED levels (reference: no CED) with reperfusion classified into successful versus unsuccessful.

| Parameters remaining after elimination | CED level versus no CED † | | | | | |
| --- | --- | --- | --- | --- | --- | --- |
|  | Mild CED | | Moderate CED | | Severe CED | |
|  | RR (95 % CI) * | P † | RR (95 % CI) * | P † | RR (95 % CI) * | P † |
| **Successful reperfusion** | **0.87 (0.73-1.02)** | **0.10** | **0.47 (0.38-0.58)** | <.05 | **0.46 (0.37-0.57)** | <.05 |
| Age, years | 1.00 (0.99-1.00) | 0.29 | 0.99 (0.98-1.00) | <.05 | 0.98 (0.98-0.99) | <.05 |
| Male sex | 1.15 (0.94-1.30) | <.05 | 1.07 (0.88-1.29) | 0.49 | 0.86 (0.71-1.05) | 0.19 |
| Oral antidiabetic medication | 1.12 (0.94-1.34) | 0.12 | 1.44 (1.09-1.79) | <.05 | 1.36 (1.07-1.73) | <.05 |
| NIHSS baseline | 1.05 (1.04-1.06) | <.05 | 1.07 (1.06-1.09) | <.05 | 1.09 (1.07-1.11) | <.05 |
| Early signs of infarction | 1.40 (1.24-1.59) | <.05 | 2.04 (1.69-2.46) | <.05 | 1.92 (1.57-2.34) | <.05 |
| Mean arterial pressure at baseline, mmHg | 1.00 (1.00-1.00) | 0.46 | 1.00 (1.00-1.01) | 0.18 | 1.01 (1.00-1.01) | 0.07 |
| Plasma glucose at baseline, mmol/l | 1.00 (0.98-1.03) | 0.10 | 1.02 (0.99-1.05) | 0.30 | 1.05 (1.03-1.08) | <.05 |
| Stroke onset to end of thrombectomy, minutes |  |  |  |  |  |  |
| ≤212 | Reference | <.05 | Reference | <.05 | Reference | <.05 |
| 213-280 | 1.38 (1.12-1.70) |  | 1.35 (0.98-1.87) |  | 1.42 (0.97-2.08) |  |
| 281-370 | 1.66 (1.36-2.04) |  | 1.28 (0.93-1.76) |  | 1.66 (1.16-2.37) |  |
| ≥370 | 1.63 (1.33-2.00) |  | 1.37 (1.00-1.89) |  | 2.01 (1.41-2.86) |  |
| Vascular territory |  |  |  |  |  |  |
| Left | Reference | <.05 | Reference | <.05 | Reference | <.05 |
| Right | 1.17 (1.03-1.33) |  | 1.43 (1.18-1.73) |  | 1.36 (1.11-1.66) |  |
| Bilateral | 1.57 (0.96-2.56) |  | 3.03 (1.68-5.48) |  | 2.49 (1.10-5.66) |  |

* Wald´s method

† LR test

**Table III**

Detailed data for the demonstration of effect modification by potential indicators of large infarct:
SHS (Severe Hemispheric Syndrome) at baseline and P-SHS (Persistent Severe Hemispheric Syndrome) at 24 hours,

using the final adjusted model.

| Successful reperfusion | SHS  (n=4541 patients included in analysis) | | | | P-SHS  (n=4168 patients included in analysis) | | | |
| --- | --- | --- | --- | --- | --- | --- | --- | --- |
|  | No | | Yes | | No | | Yes ‡ | |
|  | Cases*/n | RR (95% CI) | Cases*/n | RR (95% CI) | Cases*/n | RR (95% CI) | Cases*/n | RR (95% CI) |
| No | 59/279 | 1 | 136/377 | 0.91 (0.68-1.22) | 31/250 | 1 | 127/320 | 2.56 (1.79-3.68) |
| Yes | 110/1728 | 0.32 (0.24-0.42) | 368/2157 | 0.51 (0.39-0.67) † | 127/2722 | 0.36 (0.25-0.53) | 264/876 | 2.07 (1.45-2.96) ‡ |

* Cases of moderate or severe CED

† Among patients with SHS = Yes and successful reperfusion = Yes, the RR for moderate or severe CED is 0.51/0.91 = 0.56 (95% CI 0.48-0.66).

‡ Among patients with P-SHS = Yes and successful reperfusion = Yes, the RR for moderate or severe CED is 2.07/2.56 = 0.81 (95% CI 0.69-0.95).
